# Characteristics of Ceramic Fiber Modified Asphalt Mortar

**DOI:** 10.3390/ma9090788

**Published:** 2016-09-21

**Authors:** Jiuming Wan, Shaopeng Wu, Yue Xiao, Quantao Liu, Erik Schlangen

**Affiliations:** 1 State Key Laboratory of Silicate Materials of Architectures, Wuhan University of Technology, Wuhan 430070, China; wanjm@whut.edu.cn (J.W.); wusp@whut.edu.cn (S.W.); 2 Micromechanics Laboratory, Faculty of Civil Engineering and Geosciences, Delft University of Technology, Stevinweg 1, 2628 CN Delft, The Netherlands; H.E.J.G.Schlangen@tudelft.nl

**Keywords:** asphalt binder, asphalt mortar, ceramic fiber, dynamic shear rheometer, SuperPave rutting parameter, SuperPave fatigue parameter

## Abstract

Ceramic fiber, with a major composition of Al_2_O_3_ and SiO_2_, has advantages of stability at relatively high temperature, big specific surface area and resistance to external mechanical vibration. It has the potential contribution of improving the rutting resistance and temperature sensitivity of modified asphalt binder by proper modification design. In this research, ceramic fiber was introduced into both pen 60/80 and pen 80/100 asphalt binder by different weight ratios. An asphalt penetration test, softening point test, ductility test and dynamic viscoelastic behavior were conducted to characterize and predict the ceramic fiber modified asphalt mortar (CFAM). Research results indicated that the ceramic fiber has a great effect on reinforcement of asphalt, which makes the asphalt stiffer so that the asphalt can only undertake less strain under the same stress. The heat insulation effect of the ceramic fiber will improve the temperature stability. Complex modulus and phase angle results indicate that the ceramic fiber can significantly enhance the high temperature resistance of soft binder.

## 1. Introduction

Asphalt is the basic material of asphalt pavement, which has been used as universal pavement material on high-class pavement [1]. Asphalt pavement is generally considered to have the following advantages: high driving comfort, obvious noise reduction, resistance to skidding. However, high temperature rutting [2,3,4] and low temperature brittle fracture [5,6,7] occur frequently during pavement service life. The major cause of these distresses is the temperature sensitivity of asphalt binder, especially under repeated heavy load from vehicles.

Thus, plenty of research has been conducted to develop asphalt mixture and asphalt binder with enhanced temperature resistance, such as polymer modified asphalt, fiber reinforced asphalt concrete (FRAC) and fiber modified asphalt binder [1,8,9,10]. Fiber modification is attracting a growing amount of attention for its potential improving effects.

FRAC pavement contains all of the pavement forms [11]: Asphalt concrete (AC), Stone Matrix Asphalt (SMA), and Open Graded Friction Course (OGFC). Previous trails [12,13,14,15] have conducted a great number of FRAC experiments, and various kinds of fibers have been tested, including Polypropylene fiber, Polyester fibers, Asbestos (mineral) fiber, Cellulose fiber, Carbon fiber, Glass fiber, Nylon fiber, and Steel fiber. According to the testing results, FRAC showed its superior men mechanical properties [16] in various aspects, particularly in high temperature rutting resistance. Modification is one approach taken to improve asphalt pavement performance [17].

Ceramic fiber [18,19,20,21] is an industrial production. It has wide application value in daily life and makes up an entire industry as a material for heat insulation. Some types of ceramic fiber can even withstand the high temperature of 1600 degrees without melting or chemical reaction. The major chemical composition of ceramic fiber is Al_2_O_3_ and SiO_2_. Meanwhile, according to different purposes, some other chemical substances are added to improve its properties. The diameter of ceramic fiber ranges from 2 to 5 μm and the length is around twenty mm.

Its advantages include being lightweight, having thermal insulation, no toxicity, resistance to mechanical vibration, and being low cost compared to other fibers. In previous studies, little research on ceramic fiber modified asphalt or FRAC have been studied and the effect of adding ceramic fiber into asphalt binder is also unknown.

In order to verify the temperature susceptibility and viscoelastic effect of ceramic fiber modified asphalt mortar, a series of trials were conducted and analyzed with pen 60/80 and pen 80/100 asphalt binder in this research. An asphalt penetration test, softening point test, ductility test and Dynamic Shear Rheometer (DSR) test have been conducted. Based on the research data, the effect on high temperature rutting resistance (penetration, softening point and SuperPave rutting parameter) and low temperature cracking resistance (ductility and SuperPave fatigue parameter) of asphalt mortar can be concluded.

## 2. Experiment Materials

### 2.1. Ceramic Fiber

A 1260 °C ceramic fiber (QiangWei factory, Huzhou, China) was used in this study. The other basic index of the fiber is presented in Table 1.

**Figure 1:**
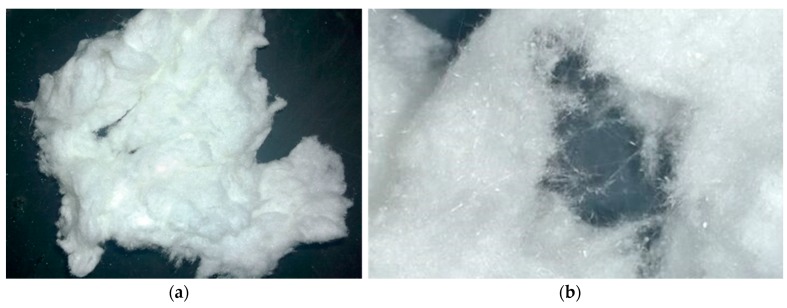
Ceramic fiber used in this research ((**a**) cluster view; (**b**) closer view).

Figure 1 presents the flocculated ceramic fiber. It is promised that such fiber has extensive flexibility under bending and torque conditions.

**Figure 2:**
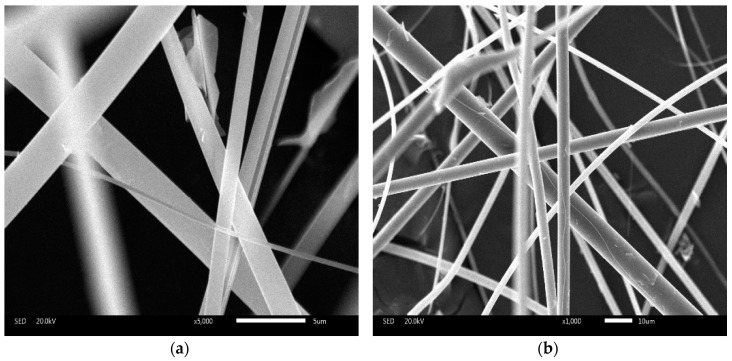
The SEM microstructure of ceramic fiber ((**a**) ×5000; (**b**) ×1000).

The surface morphology of ceramic fiber was characterized by Scanning Electron Microscopy (SEM) and the results are shown in Figure 2. The interfacial property in ceramic fiber modified asphalt is critical to its overall mechanical behavior. The SEM images indicate that the investigated ceramic fiber has a smooth surface. On the one hand, such a smooth surface will not require additional asphalt binder when it is added into asphalt mixture. On the other hand, the smooth surface will result in poor cohesive strength between binders and fibers themselves.

### 2.2. Asphalt

Two kinds of asphalt binder, pen 60/80 and pen 80/100 asphalt produced by the GuoChuang Company (Huzhou, China) were used. The fundamental properties of these binders will be presented in the data analysis along with the behavior of ceramic modified asphalt.

### 2.3. Ceramic Fiber Modified Asphalt Mortar

Normally, fibers were directly added into asphalt mixture in the asphalt plant to improve its pavement performance. In this research, fibers were considered as additives in asphalt mortar, which should include asphalt binder, fine aggregate and filler, etc. Thus, in this research, it is defined as ceramic fiber modified asphalt mortar (CFAM), although the fiber was added into asphalt binder instead of added into asphalt mixture. The mortar behavior is the core contribution to the final performance of asphalt mixture. Therefore, only rheological of CFAM was analyzed in this research.

Asphalt binder was first heated up to 150 °C till it becomes liquid. Then, a corresponding amount of ceramic fiber was slowly and gradually added, while an agitator with a stirring rotor with a leaf shape was applied at the same time. Mixing temperatures of 155 °C and 150 °C were used for pen 60/80 and 80/100 asphalt binders, respectively, due to their physical differences. The mixing time was 30 min.

**Figure 3:**
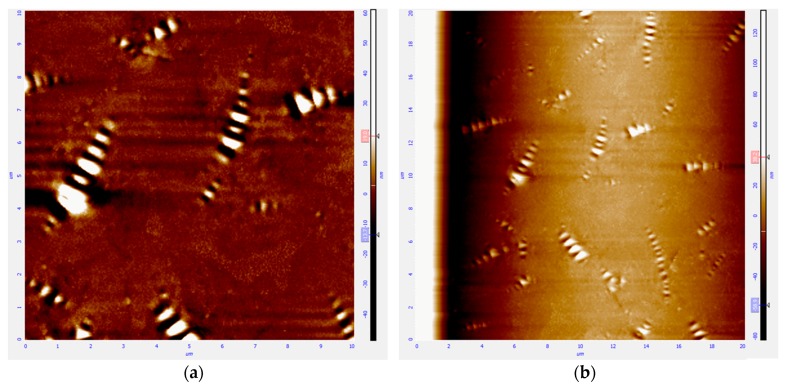
AFM scanning of ceramic fiber modified asphalt mortar.

Before conducting the scheduled physical property evaluation, homogeneous dispersion of fiber is required in the sample preparation process of CFAM. Atomic Force Microscope (AFM) was used to characterize the distribution of ceramic fiber in asphalt mortar. The AFM result of ceramic fiber in pen 60/80 asphalt is shown in Figure 3.

The left images illustrate the distribution in an area of 10 × 10 square micrometers, while the right image shows the distribution in an area of 20 × 20 square micrometers. The shining pots in AFM images are defined as bee structures with length of 2–3 microns and width less than 1 micron, which are composed of wax. Bee structure was first described by Loeber in 1996 to represent a main feature of short span concentrated in an elliptical fashion in asphalt binder at the nanoscale. The expected ceramic fiber did not show up in the AFM images, although its volume is much bigger than that of bee structures. Firstly, this means that the ceramic fiber is well covered with bitumen binder. Secondly, bad phenomena such as coalescence of fiber did not happen, since, when coalescence of ceramic fiber happens in the modified asphalt binder, the agglomeration phenomenon might display in the AFM images. The homogenous distribution of bee structures will also be influenced as well. It can be therefore be concluded that the fiber has been distributed homogenously.

The diversity of ceramic fiber in modified asphalt was carefully studied to figure out the effect on different asphalt, and then determine the application potentials of ceramic fiber in asphalt materials. Thus, different amounts of ceramic fiber, 1 wt %, 2 wt %, and 3 wt %, was added into asphalt binder.

## 3. Research Methods

This study focused on the fundamental properties of ceramic fiber modified asphalt mortar. Although conventional methods such as penetration [22], ductility [23] and ring and ball softening point [24] may still adequately describe the rheological characteristics of conventional asphalt binder, they have limited ability to accurately predict the relative performance of modified asphalt binder. Literature suggests that the dynamic shear rheometer is a powerful tool to study the rheological characteristics [25]. Therefore, five kinds of experimental tests were conducted: asphalt penetration test, softening point test, ductility test, and dynamic viscoelastic by means of DSR.

Asphalt binder is a temperature sensitive material. At low temperature or low loading duration, asphalt binder has elastic behavior, while at higher temperature or long loading duration, viscous behavior dominates. During environmental conditioning temperatures, viscoelastic behavior is observed in asphalt binder. This means that asphalt binder behaves with both elastic and vicious characteristics.

**Figure 4:**
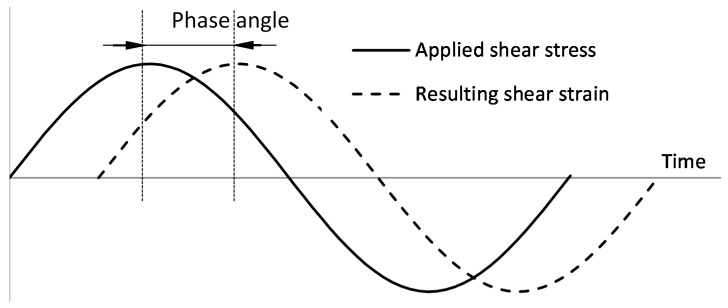
DSR applied stress, resulting strain and phase angle.

Complex modulus and phase angle are two main indexes of rheological properties for asphalt binder. The ability of asphalt resistance deformation is characterized by the complex modulus [8]. The phase angle is the lag between the applied shear stress and the resulting shear strain during sinusoidal loading (see Figure 4). It determines the viscosity and elasticity of the material, and higher value of phase angle means that the material is more close to the pure viscous material. When the δ (symbol of phase angle) is 0°, the material can be regarded as a complete elastic material. When the δ is 90°, the material can be considered as a complete viscous material.

Temperature sweep tests on asphalt are divided into two parts: higher temperature condition and lower temperature condition. The temperature of higher temperature ranges from 30 to 80 °C, while the lower temperature ranges from −10 to 30 °C. The result of this experiment can be used to predict the rheological behavior of asphalt in the actual pavement condition.

Strain control mode with a constant torque was applied, and the speed of the rotor (ω) is 10 rad/s. Strain sweep tests have been first conducted to detect the linear viscoelastic (LVE) range. The applied strain (γ), which is in the LVE range, is 0.5% in higher temperature test and 0.05% in lower temperature test. The diameter of DSR geometry in higher temperature range is 25 mm and in lower temperature range it is 8 mm, while the gap is 1 mm and 2 mm, respectively.

Groups of samples are prepared for this test. Repeated tests are conducted to ensure the reliability of the test result.

When exposed in an environment of high temperature, asphalt mixture is considered fail in rutting. Thus, asphalt mixture should have high complex modulus and low phase angle to prevent it from permanent deformation, whereas, in a low temperature condition, the major damage of asphalt is cracking. Thus, high phase angle is required to ensure better cracking resistance.

## 4. Results and Discussion

### 4.1. Basical Asphalt Properties

**Figure 5:**
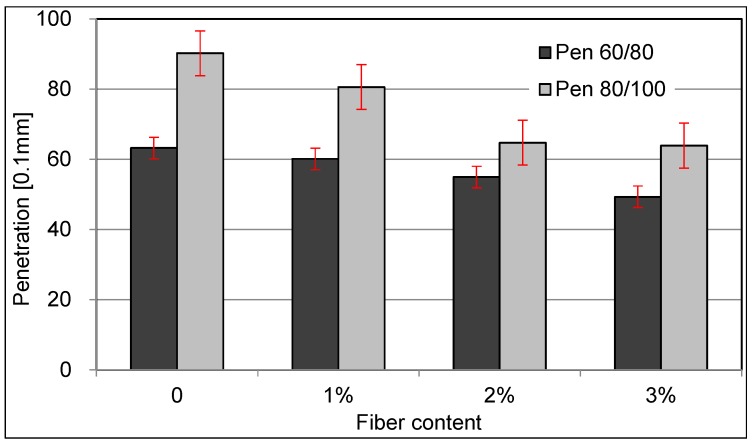
Relationship between fiber content and penetration values.

Figure 5 is the statistical results of penetration test for asphalt binder and CFAM with different fiber proportions. With the incorporation of ceramic fibers, the penetration of modified asphalt is greatly reduced. With the increase of fiber content, the decline rate of penetration has become increasingly slow. This shows that, with addition of fiber, the softness of the asphalt drops seriously. It can be noted that the fiber has a great effect on reinforcement for the asphalt, which makes it more rigid so that asphalt can only undertake less strain under the same stress.

**Figure 6:**
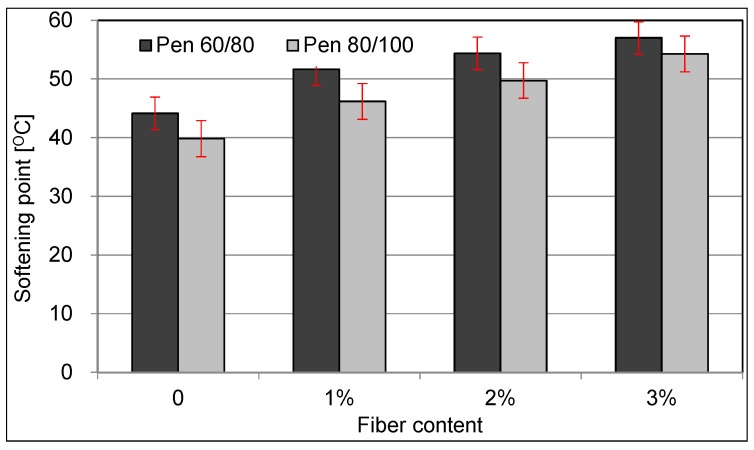
Relationship between fiber content and softening point.

Figure 6 illustrates the results of softening point test. Softening point means the temperature when the steel ball fixed on the asphalt cylinder contact the metal bottom, which is caused by the deformation of asphalt induced by the temperature change. To a certain extent, the temperature stability of asphalt is related to the softening point. When comes to the result of test, the effect on increase of softening point by the addition of fiber is quite obvious, which implies the improvement of the temperature stability.

The increasing trend of softening point for modified pen 60/80 asphalt differs from the modified pen 80/100 asphalt. In the case of modified pen 60/80 asphalt, the growth rate of softening point decreases gradually with the increase of fiber content. While the growth rate of softening point for pen 80/100 modified asphalt is generally invariant.

The reasons for this phenomenon can be concluded as two aspects: reinforcement effect and heat insulation. Ceramic fiber used in this test plays a role similar to the bone for the material, and the strength is thereby promoted, which means that the deformation of asphalt will not occur under the same temperature when base asphalt will be broken by external force. At the same time, because of the heat insulation effect of the ceramic fiber, the thermodynamic constants of asphalt binder will be also changed. The asphalt is therefore less sensitive to temperature.

Studies claimed that the softening point as well as the temperature stability should remain in a reasonable range. Too high value or too low value of softening point will cause severe damage to the asphalt [26,27]. Based on the data discussed above, the growth ranges for softening point are acceptable. Hence, this promotion is considered to be advantageous for the asphalt under high temperature.

**Figure 7:**
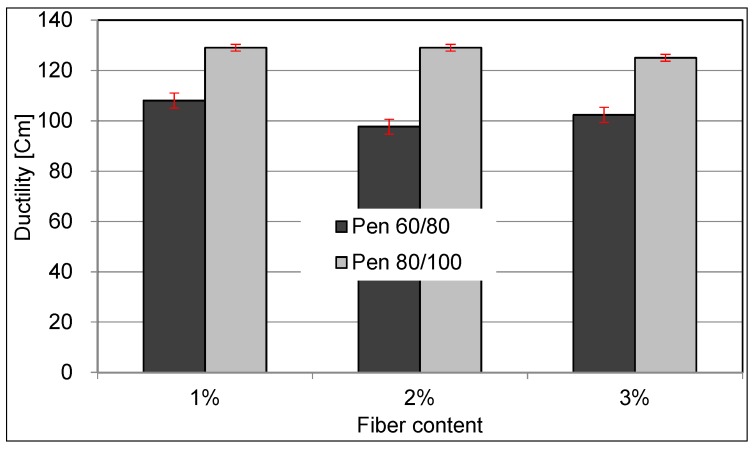
Relationship between fiber content and ductility.

The ductility value is the main index that illustrates the plasticity and ductility of the asphalt under tension conditions. The results of ductility are shown in Figure 7. The influence on the ductility resulted from ceramic fiber is negligible.

### 4.2. Rheological Properties

#### 4.2.1. Modulus and Phase Angle

**Figure 8:**
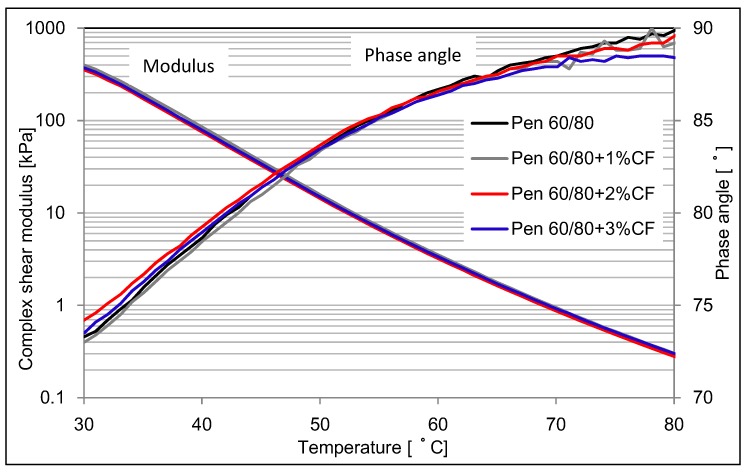
Complex modulus and phase angle of pen 60/80 based mortar at higher temperature range.

**Figure 9:**
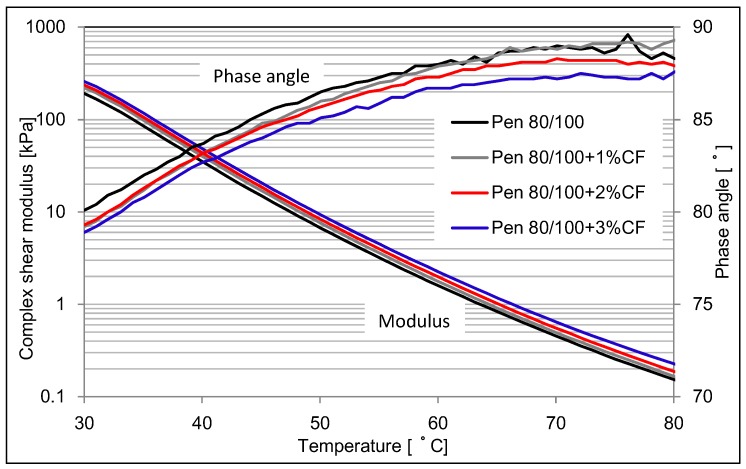
Complex modulus and phase angle of pen 80/100 based mortar at higher temperature range.

DSR was conducted to analyze the dynamic viscoelastic data for asphalt binder and CFAM. Figure 8 and Figure 9 present the complex shear modulus of asphalt binder and CFAM at higher testing temperature range.

Figure 8 shows that the studied ceramic fiber has a very limited influence on the complex modulus of pen 60/80 asphalt binder. However, Figure 9 indicates the complex modulus was significantly increased when fiber was used in pen 80/100 asphalt binder. The higher content of fiber, the higher modulus of CFAM can be achieved.

The influence on phase angle value of using ceramic fiber is similar to the influence on modulus. Ceramic fiber has a slight influence on phase angle in pen 60/80 asphalt binder, but a significant reduction in pen 80/100 asphalt binder is present. With the addition of ceramic fiber, the phase angle of modified pen 80/100 asphalt deceases relatively. The more fiber content, the lower phase angle it will be.

According to the asphalt grading specification of ASTM D6373 [28], which is standard specification for performance graded asphalt binder, pen 60/80 asphalt binder has a higher complex shear modulus and better high temperature resistance than that of pen 80/100 asphalt binder, which was can also be proven by Figure 8 and Figure 9. Therefore, results illustrate that the ceramic fiber cannot add additional high temperature resistance behavior in pen 60/80 asphalt binder, as it already has acceptable high temperature behavior. It might also be because the DSR cannot distinguish the influence of ceramic fiber on the high temperature resistance, since the improvement could be negligible. Pen 80/100 asphalt binder is a kind of softer binder. When ceramic fiber was used, it therefore was stiffened and its high temperature resistance could be improved.

**Figure 10:**
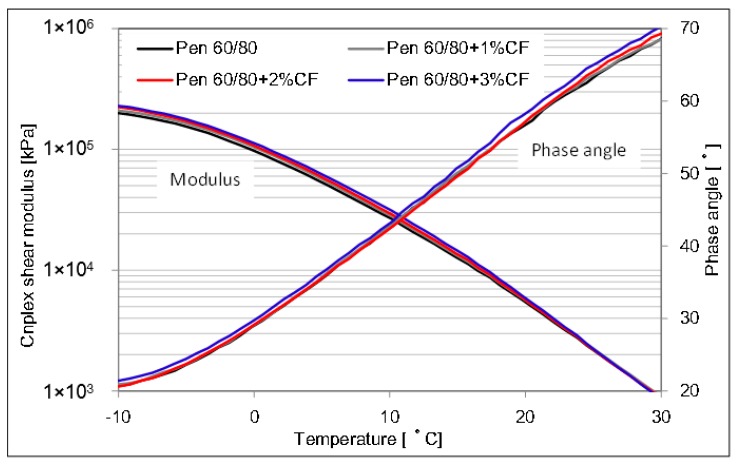
Complex modulus and phase angle of pen 60/80 based mortar at lower temperature range.

**Figure 11:**
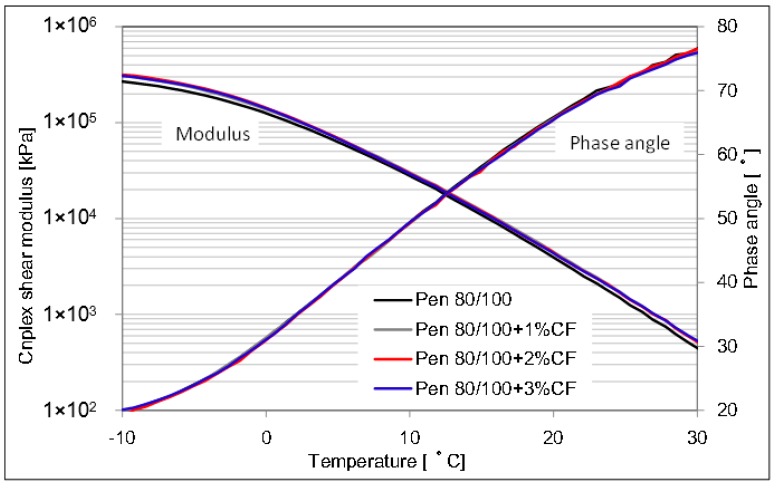
Complex modulus and phase angle of pen 80/100 based mortar at lower temperature range.

The complex shear modulus and phase angle of asphalt binder and CFAM at a lower testing temperature range are described in Figure 10 and Figure 11. The influence of additional ceramic fiber in asphalt binder shows a very limited effect on the modulus and phase angle at lower temperature range. It is difficult to determine the contribution of ceramic fiber on the low temperature behavior. Therefore, fatigue factors were studied to determine the effect of ceramic fiber on the low-temperature resistance property.

#### 4.2.2. SuperPave Rutting Parameter

According to the asphalt binder specifications resulting from Strategic Highway Research Program (SHRP), the major distress modes occurred in asphalt pavement can be predicted by rutting parameter and fatigue cracking parameter. Superpave rutting parameter is defined as the ratio between shear complex modulus and sine delta, which is G*/sinδ. The higher the rutting parameter is, the better the rutting resistance at high-temperature of the asphalt binder [16,17].

**Figure 12:**
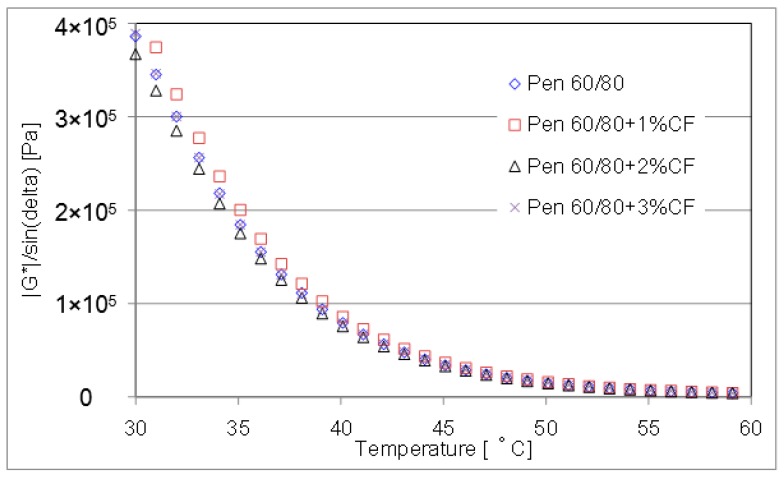
Rutting parameter of pen 60/80 based mortar at lower temperature range.

**Figure 13:**
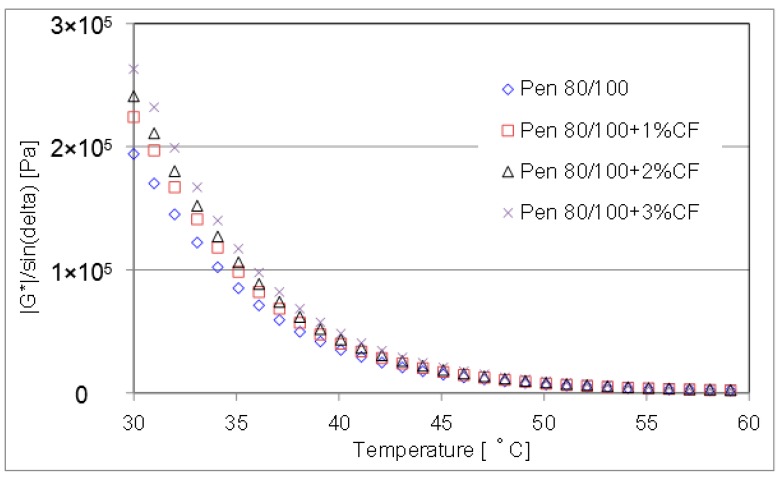
Rutting parameter of pen 80/100 based mortar at lower temperature range.

The curves of rutting parameter for evaluated CFAM were presented in Figure 12 and Figure 13. They were measured by DSR from 30 °C to 60 °C at a 20 mm depth and a frequency of 10 rad/s.

Below the test temperature of 50 °C, results show significant enhancement on the rutting resistance property was achieved when ceramic fiber was introduced. Particularly with pen 80/100 asphalt binder, the rutting parameter has been greatly improved under the contribution of using ceramic fiber. While at a temperature higher than 50 °C, the enhancement contribution of ceramic fiber is negligible.

#### 4.2.3. SuperPave Fatigue Parameter

The Superpave binder fatigue parameter, defined as G*sin δ, has been widely used as a predictor of asphalt pavement fatigue performance. The higher the fatigue parameter of asphalt binder is, the worse the fatigue resistance. According to Superpave specifications, fatigue parameter should be less than 5 MPa at 10 rad/s, at a temperature equal to the average pavement temperature in the location of interest.

**Figure 14:**
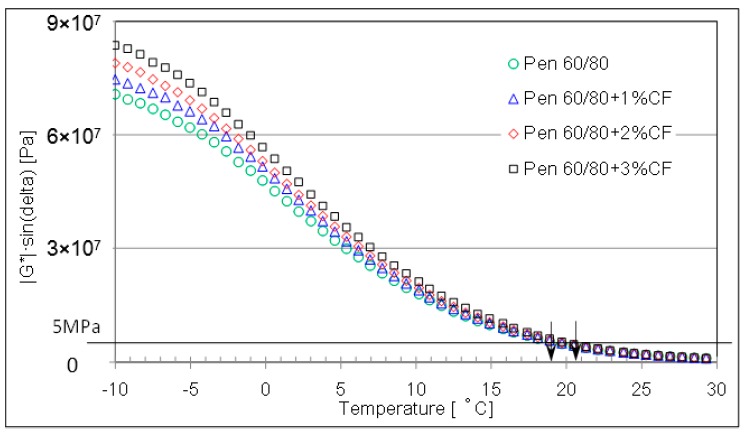
Fatigue parameter of pen 60/80 based mortar at lower temperature range.

**Figure 15:**
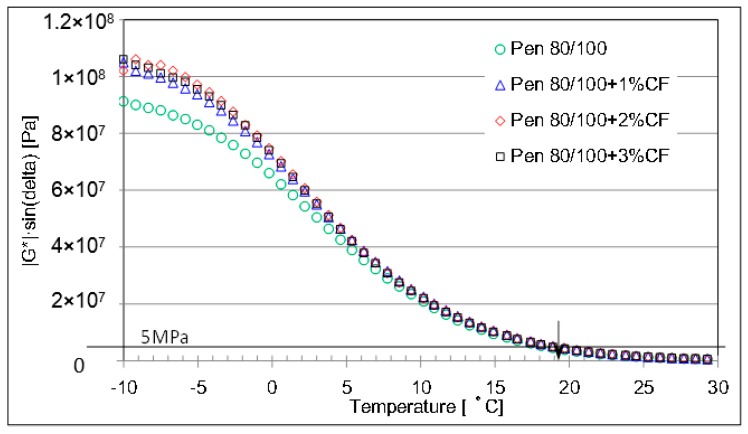
Fatigue parameter of pen 80/100 based mortar at lower temperature range.

Figure 14 and Figure 15 conclude the fatigue parameters of CFAM at lower testing temperature range. In the case of pen 60/80 asphalt binder, ceramic fiber would gradually introduce higher fatigue parameter when the temperature condition is very low. While in the case of pen 80/100, ceramic fiber increases the fatigue parameter with significant increasing ratio when the temperature is lower than 5 °C. This means that the ceramic fiber will worsen the cracking resistance of asphalt binder to some extent.

## 5. Conclusions

From the research results of ceramic fiber modified asphalt binder that were discussed in this research, the following items can be concluded:

1. The SEM images indicate that no coalescence happened in the CFAM, which means the fiber can be distributed in asphalt binder homogenously.
2. Ceramic fiber modified binder has lower penetration index, higher softening point and smaller ductility. This shows that, with the addition of ceramic fiber, the softness of the asphalt drops seriously. On the one hand, it illustrates that the fiber has a great effect on reinforcement of asphalt, which makes the asphalt stiffer so that the asphalt can only undertake less strain under the same stress. On the other hand, the heat insulation effect of the ceramic fiber will improve the temperature stability.
3. Complex modulus and phase angle results indicate that the ceramic fiber can enhance the high temperature resistance of soft binder, such as Pen 80/100 asphalt binder. In the case of hard binder, for instance pen 60/80, ceramic fiber cannot add additional high temperature resistance behavior in asphalt binder, as it already has an acceptable property of high temperature resistance.
4. Results show significant enhancement on the rutting resistance between 30 °C and 50 °C, when ceramic fiber was used to modify the asphalt binder. However, at a temperature higher than 50 °C, the influence of additional ceramic fiber is negligible.

## Figures and Tables

**Table 1:** Basic properties of the investigated ceramic fiber.

Property		Ceramic Fiber
Type		NC1260
Classification temperature (°C)		1260
Appearance		White cotton shape
Morphological structure		Glass state
Average length (mm)		2.0
Average diameter (μm)		3.0
Chemical composition (%)	Al_2_O_3_	52
	Al_2_O_3_ + SiO_2_	95
	Fe_2_O_3_	<0.2
	ZrO_2_	/
Thermal conductivity		0.03
Density (kg/m^3^)		128
